# Roles of different organizations in implementing patient-reported measures in routine maternity care in Finland

**DOI:** 10.1186/s41687-024-00793-x

**Published:** 2024-10-03

**Authors:** Kirsi Marja-Leena Väyrynen, An Chen, Seppo Heinonen, Aydin Tekay, Paulus Torkki

**Affiliations:** 1 Wellbeing Services County of Central Finland/Hospital Nova of Central Finland, Hoitajantie 3, Jyvaskyla, FI-40620 Finland; 2 Department of Public Health, Faculty of Medicine, University of Helsinki, Biomedicum 1, Haartmaninkatu 8 b, Helsinki, 00290 Finland; 3 School of Public Health, Zhejiang Chinese Medical University, No. 548 Binwen Road, Binjiang District, Hangzhou, Zhejiang Province 310053 China; 4 Department of Obstetrics and Gynaecology, Helsinki University Hospital and University of Helsinki, Haartmaninkatu 2, Helsinki, 00290 Finland

**Keywords:** Patient-centered care, Value-based healthcare, Patient-reported measures, Pregnancy and childbirth

## Abstract

**Background:**

The integration of patient-centered care (PCC) and value-based healthcare (VBHC) principles, emphasizing personalized, responsive care and cost efficiency, is crucial in modern healthcare. Despite advocation from the International Consortium for Health Outcomes Measurement (ICHOM) for the global adoption of these principles through patient-reported measures (PRMs), their implementation, especially the pregnancy and childbirth (PCB) set, remains limited in maternity care. This study focuses on understanding the optimal organizational entity for integrating standard ICHOM-PCB-PRMs into routine maternity care in Finland. It aims to clarify the distribution of tasks among stakeholders and gather Finnish maternity healthcare professionals’ perspectives on organizational responsibility in PRM collection. The emphasis was on identifying the optimal organizational framework for managing PRMs in maternity care.

**Results:**

A total of 66 maternity healthcare professionals participated in the study, reaching a consensus that public maternity care centers in Finland should be the primary entity responsible for managing PRMs in the maternity sector. Key aspects such as *confidence with the role as a mother, maternal confidence with breastfeeding*, and *satisfaction with the result of care* were identified as crucial and should be inquired about in both public maternity care centers and hospital maternity wards. The findings highlight the importance of comprehensive and consistent attention to these PRMs across public maternity care centers and hospital maternity settings to ensure holistic and effective maternal care.

**Conclusions:**

The study highlights the central role of public maternity care centers in the collection and management of PRMs within Finnish maternity care, as agreed upon by the professional consensus. It underscores the importance of a consistent and holistic approach to PRM inquiry across different care settings to enhance the quality and effectiveness of maternity care. This finding is crucial for policymakers and healthcare practitioners, suggesting that reinforcing the collaborative efforts between public maternity care centers and hospital maternity wards is vital for a patient-centric, efficient healthcare system. Aligning with PCC and VBHC principles, this approach aims to improve healthcare outcomes for pregnant and postpartum women in Finland, emphasizing the need for a unified strategy in managing maternity care.

**Supplementary Information:**

The online version contains supplementary material available at 10.1186/s41687-024-00793-x.

## Introduction

Patient-reported measures (PRMs) are powerful tools renowned for enhancing shared decision making at the individual patient level [1]. This is particularly beneficial in fields where positive clinical outcomes are prevalent, with a low occurrence of serious adverse events. In the realm of pregnancy and childbirth care, prevailing quality measures often concentrate on negative outcomes such as morbidity and mortality. However, in many high-income countries, these adverse outcomes are infrequent, and most pregnancies are anticipated to unfold smoothly from a clinical standpoint. Embracing PRMs can unlock new dimensions for enhancing the quality of care, focusing not only on preventing negative outcomes but also on maximizing positive experiences for expectant mothers.

In modern healthcare, patient-centered care (PCC) and value-based healthcare (VBHC) are vital [2–6]. PCC prioritizes individual needs, preferences, and values, emphasizing respectful and responsive care. VBHC seeks higher-quality care at lower costs [7]. The International Consortium for Health Outcomes Measurement (ICHOM) advocates for VBHC principles, developing outcome measurement sets like patient-reported outcomes (PROMS) and experiences (PREMS) in maternity care [8]. PROMS collects direct patient information, while PREMS focuses on perceptions and experiences [9–13]. Integrating PRMs with clinical outcomes empowers healthcare professionals to understand patient physical symptoms, emotions, health related quality of life (HRQoL) identify areas for improvement, and enhance communication, which are essential for realizing the goals of both PCC and VBHC [14–19]. The ICHOM pregnancy and childbirth standard set (ICHOM-PCB-PRM) encompasses a range of concerns, such as health-related quality of life, pain during intercourse, maternal role confidence, mother’s attachment to the infant, breastfeeding, postpartum depression, satisfaction with care, confidence in healthcare providers, and the birthing experience [20]. Further details about the ICHOM-PCB-PRM can be found in Additional File 1.

Some studies investigated the ICHOM-PCB set in the Netherlands, which underlined the need for local adaptations: the need for agreement of the professional’s responsibilities, well-structured ICT tools, and conclusion on how outcomes are distributed [21–24]. The ICHOM-PCB set was also translated into German and required adapting questions or making the response structures according to the local manner [25]. The empirical evidence showed that routine use of PRMs in maternity care could help detect health problems, clarify clinical visit preparation, and ease communication between professionals and women [26, 27]. Still, there might be challenges to motivate women to answer PRM questions and to motivate professionals to respond to answers, provide follow-up care, and build information systems for data collection [10, 26–28].

In the Finnish maternity care system, various healthcare providers are involved, including public maternity care centers, maternity clinics, delivery wards, and maternity wards [29]. While there are studies according to development on PRMs in maternity care, there is still a lack of knowledge of who should take responsibility in implementing them into practice [30–33].

The aim of this study is to get maternity healthcare professionals’ opinions based on their experience regarding the main responsible organization, in the field where there are many providers, on collecting the PRM battery of questions and the necessary healthcare aids regarding the issues of pregnant women. This is, as far as we know, the first study that explores the main responsible organization in the multilevel maternity care provider chain.

## Materials and methods

### Study design

The study design is a mixed-method approach to delve into the organization of maternity care and the integration of PRMs within this framework. Public care centers, supported by state funding, play a pivotal role in providing equitable and complimentary services to all expectant mothers. These centers ensure that pregnant women receive consistent check-ups, are referred for voluntary fetal screenings, and undergo comprehensive health assessments to monitor the pregnancy’s progression and identify any potential issues early on. Simultaneously, maternity clinics located within hospitals are instrumental in delivering specialized prenatal care, particularly targeting high-risk pregnancies. These clinics are equipped to offer a broad spectrum of services, including medical examinations, counseling, and access to various educational resources. It is noteworthy that, in Finland, almost all births take place within hospital delivery wards [26, 27].

In this study, maternity healthcare professionals are invited to participate in a survey featuring multiple-choice questions. This survey is designed to identify the primary organization responsible for the effective utilization of the ICHOM-PCB-PRM. Additionally, respondents can provide answers to open-ended questions, drawing upon their professional expertise regarding the subject matter. This methodological choice builds upon our previous qualitative investigations, which involved semi-structured interviews with Finnish maternity care professionals aimed at capturing their perspectives on the practical application of PRMs in maternity care settings [25]. The consensus from an earlier study highlighted the critical importance and relevance of adopting PRMs into the systematic practice of Finnish maternity care. However, these investigations did not clarify the main entity responsible for orchestrating this implementation. Therefore, the objective of the present study is to bridge this gap by exploring and identifying the key organizer(s) behind the incorporation of PRMs into the Finnish maternity care system, thus enhancing the quality and efficiency of prenatal and childbirth-related healthcare services.

### Participant recruitment

Our aim was to recruit at least 65 healthcare professionals to this study from the wellbeing services in central Finland: doctors, obstetricians in the hospital and public maternity care centers, nurses working in the public maternity care centers, screening unit midwives, and midwives working in the hospital’s delivery room and in the hospital ward. The targeted number of participants was based on the number of public maternity care center networks in the area in relation to the number of hospital staff. This way, we could have the important information from all aspects of maternity care.

We started recruiting in June 2023 and ended in October. Within that time, we gathered 66 participants (Table 1). One researcher (KV) kept two online meetings for local public maternity care center nurses to discuss the study and to recruit participants. We also sent an email to healthcare professionals, where we gave information about the study and asked for voluntary participants. One researcher (KV) also gave face-to-face presentations in the hospital ward to delivery room professionals. Being part of the study was voluntary, and written consent was obtained. We created webpages (www.proms.fi) for information. We used the web tool for interviews, which opened with a personal code. The qualitative research utilized semi-structured interviews conducted via a web tool to address real-world practical issues. This approach incorporated both multiple-choice questions and the chance to respond to open-ended questions.

### Data collection and analysis

We asked experts’ opinions on each of the ICHOM-PCB themes: *background information, health-related quality of life, incontinence, pain with intercourse, confidence with role as a mother, mother-infant attachment, maternal confidence with breastfeeding, success with breastfeeding, postpartum depression, satisfaction with the result of care, confidence as an active participant in healthcare decisions, confidence in healthcare providers*, and *birth experience* [34]. Our investigation showed that, in Finland, asking patients about their race/ethnicity for medical purposes is banned, leading us to omit this question from our study. We also excluded queries on obstetric history, as service providers can access this information through patient systems [28]. Furthermore, following Finland’s advice against giving infants under six months water, we removed “water” from the breastfeeding success metric. We asked which organization should ask these questions, which platform would be used to ask, which organization should read the answers, and which organizations should respond if help is needed. The timing of the questions was asked as well. It was optional to choose one or many options for the answer. With every question, there were options to respond to open-ended questions. A reminder to fill in the questionnaire was sent via email two weeks after the initial invitation. The estimated time to answer all questions was 30–60 min. The survey was conducted in Finnish and then KV, being proficient in both Finnish and English, translated the answers sentence by sentence from Finnish to English. Questionnaires with answers were analyzed with Excel.

## Results

### Basic characteristics of participants

The survey participants were 66 professionals from the maternity care pathway (Table 1).

**Table 1:** The main organizations that should be responsible for presenting, receiving, and responding to PRMs

Occupation	Participants *n* = 66	Years of experiences, mean, range
Nurses in public maternity care centers	17	19, 6–37
Midwives in the hospital	30	20, 1–35
Doctors in public maternity care centers and hospitals	19	10, 1–35

### Organization that should take responsibility for presenting PRMs

The consensus among most participants was that all PRMs should be asked in public maternity care centers. For specific aspects, the majority believed that certain PRMs should also be addressed in the hospital maternity ward. These included *confidence with role as a mother* (*n* = 53, 80%), *maternal confidence with breastfeeding* (*n* = 53, 80%), *satisfaction with the result of care* (*n* = 46, 70%), *confidence in healthcare providers* (*n* = 45, 68%), and *birth experience* (*n* = 50, 76%).

### Organization that should take responsibility for receiving PRMs

**Fig. 1:**
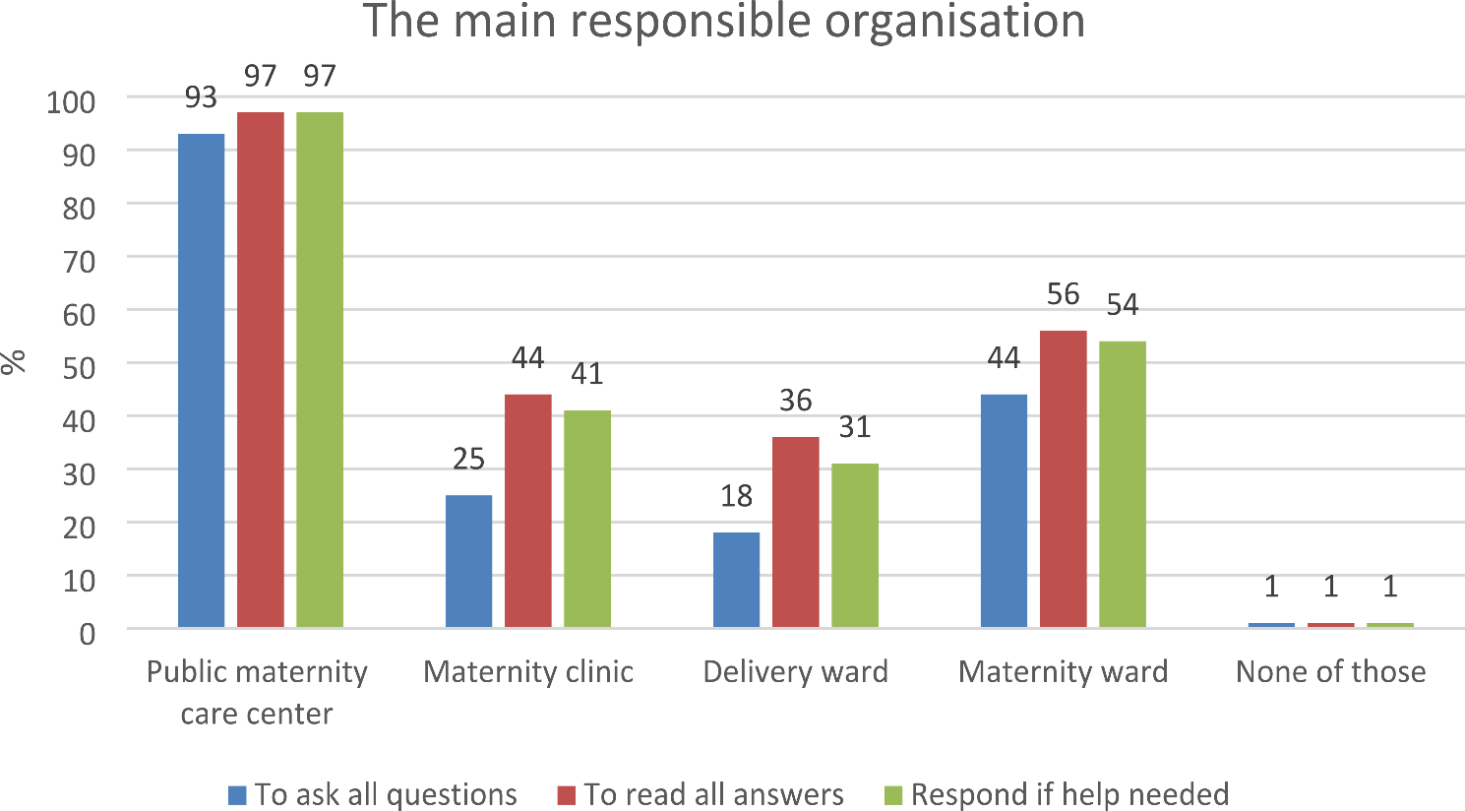
Professionals’ justification for main responsible organization for presenting, receiving, and responding to PRMs

Most participants (93%) agreed that answers to PRMs should be received in both public maternity care centers and the hospital maternity ward, while 56% supported the idea of the maternity ward (Fig. 1). *Background information* answers were deemed important to be read by all organizations, with full agreement for public maternity care centers (*n* = 66, 100%) and varying agreement for maternity clinics (*n* = 50, 76%), delivery wards (*n* = 35, 53%), and maternity wards (*n* = 38, 58%). In addition to public maternity care centers (*n* = 66, 100%), *health-related quality of life* was considered relevant in the hospital maternity clinic, with 59% agreement (*n* = 39). *Satisfaction with the result of care* was universally important to read, with full agreement for public maternity care centers (*n* = 66, 100%) and varying levels of agreement for maternity clinics (*n* = 45, 68%), delivery wards (*n* = 41, 62%), and maternity wards (*n* = 53, 80%). *Confidence as an active participant in healthcare decisions* and *confidence in healthcare providers* were both regarded as important in every organization. Agreements ranged from 55 to 97%, with only a small percentage indicating none of these (0–3%). *Birth experience* answers were highlighted as crucial to read in public maternity care centers (*n* = 50, 76%), delivery wards (*n* = 49, 74%), and maternity wards (*n* = 44, 67%).

### Organization that should take responsibility for responding to PRMs

Most participants agreed that public maternity care centers (97%) and hospital maternity wards (54%) are the organizations that should respond when help is needed. All 66 participants (100%) acknowledged the significance of addressing *health-related quality of life* at public maternity care centers. Additionally, 61% (*n* = 40) expressed the importance of addressing this issue not only in public maternity care centers but also in the hospital maternity clinic. *Incontinence* was thought to be a topic that should be responded to in public maternity care centers (*n* = 65, 98%) and in maternity clinics (*n* = 34, 52%). *Satisfaction with the care* results emerged as a key concern that should be addressed across all organizations, with high agreement in public maternity care centers (*n* = 65, 98%) and moderate agreement in maternity clinics (*n* = 38, 58%), delivery wards (*n* = 36, 55%), and maternity wards (*n* = 47, 71%). Participants emphasized the importance of fostering *confidence as an active participant in healthcare decisions*. The consensus was strong in public maternity care centers (*n* = 63, 95%) and maternity wards (*n* = 45, 68%), while maternity clinics (*n* = 40, 61%) and delivery wards (*n* = 34, 52%) expressed this need to a slightly lesser extent. *Confidence in healthcare providers* was considered essential in all organizations, with notable agreement in public maternity care centers (*n* = 63, 95%), maternity wards (*n* = 49, 74%), maternity clinics (*n* = 42, 64%), and delivery wards (*n* = 35, 53%). The significance of inquiring about the *birth experience* was highlighted, particularly in delivery wards (*n* = 46, 70%), alongside public maternity care centers (*n* = 51, 77%) and maternity wards (*n* = 42, 64%).

Participants were given an option to freely tell the reasoning for the choices they made based on their experience of the subject. Table 2 shows the majority (> 50%) of respondents regarding the place to present questions. Table 3 similarly shows the results regarding the organization that should read all PRMs. Table 4 shows the organization that should respond if help is needed and professionals’ opinions about them. Across all three tables, there is a clear indication of the central role played by public maternity care centers in the continuum of care for expectant and new mothers. These centers are seen as the primary venue for asking questions, interpreting responses, and providing necessary interventions. However, the data also support a collaborative model of care, where hospital maternity wards play an important role in specific instances, especially before discharge. The emphasis across the tables on quick and effective responses, the importance of feedback, and the need for inter-organizational communication highlight a holistic approach to maternal care. This approach prioritizes not only the physical health of mothers and infants but also their mental and emotional wellbeing. The healthcare professionals’ insights suggest a model of care that is adaptive, patient-centered, and integrated, reflecting an understanding of the complex needs of mothers and families during the prenatal and postnatal periods.

**Table 2:** Organization to ask with responders’ comments

Questions to be asked	Public maternity care center	Comments for public maternity care center	Maternity ward in hospital	Comments for maternity ward in hospital
Background information	*n* = 65, 98%	*“This is the usual background information, the collection of which during pregnancy is, in my opinion, part of the basic work of the public maternity care center.” Nurse in public maternity care center, 9 years’ experience*		
Health-related quality of life	*n* = 66, 100%	*“Public maternity care centers reach almost all expectant/birth families. Not all of those who have visited, for example, end up in the maternity unit. Of course, based on the questions and answers, you can also take those elsewhere, when the information is clearly available.” Midwife in hospital, 4 years’ experience*		
Incontinence	*n* = 64, 97%	*“After giving birth, it is important to talk about it and discuss it. Normalizing and, on the other hand, saying what is not normal. The task of the public maternity care centers is to map the recovery from childbirth and, if necessary, to consult/contact specialized medical care.” Midwife in hospital, 13 years’ experience*		
Pain with intercourse	*n* = 65, 98%	*“Often already at the family planning public care centers, the issue comes up before pregnancies, but information is important when conducting examinations so that they do not become repulsive and scary.” Nurse in public maternity care center, 37 years’ experience*		
Confidence with role as a mother	*n* = 66, 100%	*“Primarily mapping in public maternity care center, but it would be a good thing to map the situation also when leaving the maternity hospital.” Midwife in hospital, 23 years’ experience*	*n* = 53, 80%	*“Already during pregnancy, the mother-to-be and family’s capacity and support needs are mapped. The maternity ward before discharge gets some sort of picture of the family’s and mother’s resources, albeit for a short period of time.” Nurse in public maternity care center, 16 years’ experience*
Mother-infant attachment	*n* = 65, 98%	*“Usually there are no more contacts with the hospital after the baby is born.” Nurse in public maternity care center, 22 years’ experience*		
Maternal confidence with breastfeeding	*n* = 65, 98%	*“The survey should be carried out for the first time already during pregnancy and repeated after the birth so that the need for special breastfeeding support can be identified and treated.” Midwife in hospital, 13 years’ experience*	*n* = 50, 76%	*“Supporting breastfeeding and owning one’s decision belongs to everyone.” Midwife in hospital, 30 years’ experience*
Success with breastfeeding	*n* = 66, 100%	*“At this stage, usually within the public maternity care center. The mother no longer visits the maternity clinic or the ward.” Doctor, 6 years’ experience*		
Postpartum depression	*n* = 66, 100%	*“In my opinion, these questions belong to the scope of the post-examination, in the public maternity care center.” Nurse in public maternity care center, 2 years’ experience*		
Satisfaction with the result of care	*n* = 64, 97%	*“This could be given from the maternity ward and returned at the follow-up examination in the public maternity care center, important feedback for the entire care chain.” Doctor, 15 years’ experience*	*n* = 46, 70%	*“Differentiated pregnancy, childbirth, and postpartum care; that is, every organization would ask its own part.” Nurse in public maternity care center, 37 years’ experience*
Confidence as an active participant in healthcare decisions	*n* = 63, 95%	*“Such a survey could be made in the public maternity care center with follow-up if needed in a place where things went wrong.” Nurse in public maternity care center, 16 years’ experience*	*n* = 39, 59%	*“Feedback questions for each organization.” Nurse in public maternity care center, 37 years’ experience*
Confidence in healthcare providers	*n* = 64, 97%	*“The public maternity care center is the provider that meets the family after the birth.” Nurse in public maternity care center, 19 years’ experience*	*n* = 45, 68%	*“Especially if there was something special during the birth, such a small conversation after the birth could be in order.” Nurse in public maternity care center, 12 years’ experience*
Birth experience	*n* = 43, 65%	*“In the follow-up examination in the public maternity care center.” Doctor, 6 years’ experience*	*n* = 50, 76%	*“Questionnaire in the maternity ward before discharge and in the public maternity care center at the time of the follow-up examination. You can’t ask in the delivery ward; it is too recent.” Doctor, 18 years’ experience*

**Table 3:** Organization to read the answers with responders’ comments

Answers to be read	Public maternity care center	Comments for public maternity care center	Maternity ward in hospital	Comments for maternity ward in hospital
Background information	*n* = 66, 100%	*“Public maternity care centers primarily use data, but the maternity clinic/hospital also has access to data if, for example, there is a change of hospital or concerns arise about the mother’s situation.” Midwife, 28 years’ experience*	*n* = 38, 58%	*“Important information for all.” Midwife, 26 years’ experience*
Health-related quality of life	*n* = 66, 100%	*“Public maternity care centers could do the first visit, etc. and contact to ask these and read the answers. Others should also have the opportunity to update information in the future.” Nurse in public maternity care center, 16 years’ experience*		
Incontinence	*n* = 66, 100%	*“Urinary incontinence during pregnancy and after childbirth is common. The normalization of these, and the generality, on the other hand, supports the role of public maternity care centers in asking questions.” Doctor, 6 years’ experience*		
Pain with intercourse	*n* = 66, 100%	*“The matter should be clarified at the beginning in primary health care.” Doctor, 22 years’ experience*		
Confidence with role as a mother	*n* = 66, 100%	*“Everyone is responsible for supporting early interaction.” Nurse in public maternity care center, 8 years’ experience*	*n* = 52, 79%	*“Important for everyone to know. To know how to allocate sufficient help and support and guidance to everyone.” Midwife, 4 years’ experience*
Mother-infant attachment	*n* = 66, 100%	*“Of course, it depends on where in the pregnancy/after the birth the question is asked. But it would be good to know the thoughts of the mother/family of the caregivers regarding the baby.” Midwife, 4 years’ experience*		
Maternal confidence with breastfeeding	*n* = 65, 98%	*“If there were a comprehensive set of questions about breastfeeding online, it could be used in different units depending on the situation.” Midwife, 23 years’ experience*	*n* = 55, 83%	*“The maternity ward can immediately pay attention to possible future challenges.” Doctor, 6 years’ experience*
Success with breastfeeding	*n* = 66, 100%	*“This is postpartum information.” Nurse in public maternity care center, 37 years’ experience*		
Postpartum depression	*n* = 66, 100%	*“The mental health of the expectant mother is important for everyone taking care of pregnancy and childbirth to know.” Nurse in public maternity care center, 15 years’ experience*		
Satisfaction with the result of care	*n* = 66, 100%	*“Public maternity care centers primarily, but if something special arises from the answers, then the patient should be guided to the hospital.” Doctor, 6 years’ experience*	*n* = 53, 80%	*“It would be good for each unit to know how satisfied patients have been with the care chain at any stage.” Nurse in public maternity care, 19 years’ experience*
Confidence as an active participant in healthcare decisions	*n* = 61, 92%	*“Development of the unit’s and individuals’ operations and, of course, also feedback on things that went well are important.” Midwife, 4 years’ experience*	*n* = 50, 76%	*“Important feedback for everyone.” Doctor, 15 years’ experience*
Confidence in healthcare providers	*n* = 64, 97%	*“Feedback is always important for the organization in order to make changes in operations if necessary.” Midwife, 20 years’ experience*	*n* = 53, 80%	*“Feedback from all levels, but it’s not worth asking everywhere separately.” Doctor, 15 years’ experience*
Birth experience	*n* = 50, 76%	*“Public maternity care centers, if you think about the need for the next birth, to prevent the fear of birth.” Nurse in public maternity care centers, 37 years’ experience*	*n* = 44, 67%	*“This is a measure of treatment quality and treatment satisfaction.” Midwife, 28 years’ experience*

**Table 4:** Organization to respond if help is needed with responders’ comments

Item responded to	Public maternity care center	Comments for public maternity care center	Maternity ward in hospital	Comments for maternity ward in hospital	How these should be responded to
Health-related quality of life	*n* = 66, 100%	*“Public maternity care centers have a broader overall picture of the patient’s state of health and need for help. The maternity hospital focuses more on the monitoring and treatment of pregnancy and childbirth. The public maternity care center reports, consults, and, if necessary, sends them to the hospital.” Midwife, 15 years’ experience*			*“Worrying answers should be considered as soon as possible, and help should be planned in cooperation with the patient (social work if necessary). Reporting the action on paper is important so that several people do not handle the same thing.” Midwife, 28 years’ experience*
Incontinence	*n* = 65, 98%	*“The public maternity care center has an idea of what a normal pregnancy-related problem is and what is not.” Midwife, 26 years’ experience*			*“The public maternity care center can also react if these come up at the first visit. Diet/exercise/pelvic floor instructions at least. Maternal health care provider’s reaction in the future. Degree of harm? Need for a pelvic floor unit?” Midwife, 20 years’ experience*
Pain with intercourse	*n* = 64, 97%	*“The matter should be clarified at the beginning in primary health care.” Doctor, 22 years’ experience*			*“If necessary, refer to a doctor or sexual health unit. A sexual counselor can also visit the reception at the same time as the mother, if the mother gives her permission.” Nurse in public maternity care center, 20 years’ experience*
Confidence with role as a mother	*n* = 66, 100%	*“If the parent feels that they need help, then they should try to support this. Usually quickly after returning home, the responsibility falls very much on the public maternity care centers.” Midwife, 2 years’ experience*	*n* = 57, 86%	*“Primarily public maternity care center, but if the situation occurs before discharge in the hospital, then the matter should also be reacted to in the hospital.” Midwife, 23 years’ experience*	*“If necessary, the organization of additional support*, e.g.,* infant psychiatric working group.” Midwife, 23 years’ experience*
Mother-infant attachment	*n* = 66, 100%	*“Primarily, these issues come up in the public maternity care center after the birth, but sometimes support and help for the mother’s situation must be arranged already in the hospital.” Midwife 23 years’ experience*			*“Help must be arranged as soon as possible so that the situation does not get worse.” Midwife, 30 years’ experience*
Maternal confidence with breastfeeding	*n* = 64, 97%	*“Refer from the public maternity care center to the breastfeeding unit in the maternity clinic. The treatment considers when the mother is about to give birth.” Midwife, 28 years’ experience*	*n* = 56, 85%	*“The best know-how when it comes to breastfeeding: maternity ward.” Doctor, 35 years’ experience*	*“Giving guidance on uncertain issues, observing the breastfeeding situation, offering evidence-based information, or booking an appointment at the breastfeeding unit.” Midwife, 2 years’ experience*
Success with breastfeeding	*n* = 66, 100%	*“Breastfeeding is an individual thing, support individually.” Nurse in public maternity care center, 25 years’ experience*			*“Ask in more detail if the mother does not fully breastfeed, why this is the case, if there are any problems, if she wants to breastfeed, etc. and guide her in breastfeeding or send her to a breastfeeding unit to a consultant if necessary.” Midwife, 20 years’ experience*
Postpartum depression	*n* = 66, 100%	*“The mental health of the expectant mother is important for everyone caring for pregnancy and childbirth to know.” Nurse in public maternity care center, 15 years’ experience*			*“Psychologist services in the public maternity care center, doctor’s appointment if necessary.” Midwife, 2 years’ experience*
Satisfaction with the result of care	*n* = 65, 98%	*“Depending on the situation, it is important that someone reacts.” Midwife, 4 years’ experience*	*n* = 47, 71%	*“In accordance with the organization’s feedback practices. Supervisors first and then, if necessary, feedback to employees.” Doctor, 35 years’ experience*	*“Take feedback to the staff and fix things that need improvement. Handle feedback in staff meetings.” Midwife, 2 years’ experience*
Confidence as an active participant in healthcare decisions	*n* = 63, 95%	*“You can also send feedback to the maternity clinic or delivery ward. Results are more reliable if an outside party asks or can answer anonymously.” Doctor, 35 years’ experience*	*n* = 45, 68%	*“If there is frequent dissatisfaction, you should think about the cause and the need to change.” Doctor, 6 years’ experience*	*“The results of treatment satisfaction surveys should be processed in organizations on a unit-by-unit basis, and efforts should be made to improve treatment satisfaction with concrete means.” Nurse in public maternity care center, 21 years’ experience*
Confidence in healthcare providers	*n* = 63, 95%	*“Especially the one organization that has had problems.” Midwife, 26 years’ experience*	*n* = 49, 74%	*“The maternity ward may need to organize a follow-up discussion, etc. Public maternity care centers respond to its feedback.” Doctor, 22 years’ experience*	
Birth experience	*n* = 51, 77%	*Follow-up discussion with the midwife and doctor if you feel the birth experience was bad. The public maternity care center could also consult*, e.g.,* a psychologist if the client needs longer-term help to go through the experience.” Midwife, 20 years’ experience*	*n* = 42, 64%	*“The after-discussion with those who took care of the birth would be good. It may be more difficult for nurses working in public maternity care centers to help in this matter, but of course a referral can be made to the hospital for an after-care discussion.” Nurse in public maternity care center, 12 years’ experience*	*“Offer the opportunity to talk with the people who took care of the birth.” Midwife, 2 years’ experience*

PRMs intended for presentation, reception, or response across all organizations are PREMs. These include assessing *satisfaction with the result of care, shared decision making, confidence in care providers*, and *birth experience*. Participants, through answering open-ended questions, underscored the importance of feedback and a deliberate opportunity for enhancing working methods within all organizations.

The results indicate a consensus among participants regarding the importance of addressing various PRMs in both public maternity care centers and hospital maternity settings. Key aspects such as *confidence with role as a mother, maternal confidence with breastfeeding, satisfaction with the result of care, confidence in healthcare providers*, and *birth experience* were identified as crucial and should be inquired about in both public maternity care centers and hospital maternity wards. Additionally, *background information* and *health-related quality of life* were considered important across organizations, with unanimous agreement on public maternity care centers. *Satisfaction with the care* results and fostering *confidence as an active participant in healthcare decisions* were also highlighted as significant topics to be addressed in all organizations, although agreement varied across different settings. Participants expressed a strong consensus that public maternity care centers and hospital maternity wards are the primary organizations that should respond if help is needed, with unanimous acknowledgment of the significance of addressing *health-related quality of life* in public maternity care centers. *Incontinence* was identified as a topic to be responded to by public maternity care centers and maternity clinics. Overall, the findings emphasize the importance of comprehensive and consistent attention to various PRMs across public maternity care centers and hospital maternity settings to ensure holistic and effective maternal care.

## Discussion

Expectant mothers receive complimentary maternity care through the national healthcare and social welfare system in Finland [29]. The objective is to ensure that all women have access to equitable and safe care throughout the stages of pregnancy and childbirth. Nevertheless, we lack systematically collected PRMs in maternity care, which could empower healthcare professionals to uncover hidden health issues, comprehensively monitor a patient’s health status over time, assess service impact, gauge clinical relevance for patients, and improve communication [14–16]. While Finnish healthcare professionals and researchers acknowledged the potential significance of PRMs in enhancing information, improving services and care, making managerial advancements, and developing the maternity care system [28], the interviewees specifically pointed out shortcomings in the fragmented service system. They criticized the lack of effective collaboration between key providers, namely public maternity care centers and hospitals, leading to inadequately organized information sharing and transfer. There is need to intensive work, education, and resources before achieving the goal. However, most professionals believe that introducing PRMs would streamline the integration process. The delivery of integrated and continuous care has been highlighted as a significant objective by the Finnish authorities [35].

In our earlier study, women were asked to give their opinion of the ICHOM-PCB-PRM set [10]. Overall, the participants believed that by systematically applying PRMs in maternity care pathways, their needs and intentions could be better heard by the professionals. They thought that PREMS should be asked repeatedly during pregnancy and by different healthcare service providers, underlying the fact that maternal health status and experiences change over time. Participants pointed out the importance of responses and follow-up care if needed. There were also some concerns about answering honestly if the follow-up actions were unclear. There were suggestions to answer easy and short questions digitally before appointments. As we noted, due to the involvement of various providers in the public maternity care system in Finland, it was imperative to establish clear definitions for the responsibility and procedures associated with data collection, policies governing data sharing and integration, and the distribution of tasks for follow-up actions based on PRMs, as we did with this current study.

Several studies on PROMs have been published from the Netherlands [11, 21, 22, 32, 36]. One of those studies was an extensive collaborative effort conducted across seven obstetric care networks [32]. This approach involved both hospital-based obstetric teams and community midwives working together to integrate PCB-PRMs into routine clinical practice. In contrast to our study, the primary objective was not to delineate the main healthcare provider in perinatal care but to enrich the perinatal care model through the implementation and evaluation of PRMs. This multidisciplinary project underscored the collaborative nature of perinatal care, highlighting the vital roles played by both hospitals and midwives in providing comprehensive care. The focus was squarely on how patient-reported measures could be effectively integrated into clinical workflows to improve the quality of care. The inclusive approach across various healthcare providers within the obstetric care networks emphasized the study’s goal of enhancing personalized care and shared decision making, rather than identifying a main provider. Through this collaborative model, the study aimed to demonstrate the benefits of integrating patient feedback into clinical practice, ultimately advancing the transformation toward patient-centered, value-based health care in perinatal services. As far as we know, our study is the first one aiming to find out the main healthcare provider that should be responsible for collecting and handling PRMs in maternity care.

Before actual implementation of PRMs into systematic use in maternity care, we needed to explore the options from healthcare providers. That is why we built this mixed-method survey study among experts: nurses, midwives, and doctors in public maternity care centers and delivery hospitals. We conducted this study in the wellbeing services county of central Finland because of its considerable size, being the largest non-university hospital, and its significant role in maternity care. In 2021, there were 2127 deliveries in the catchment area of the hospital. All fetal screening of pregnant women living in the area has been provided by the hospital. In 2020, 87% of pregnant women participated in early pregnancy screening and 94% in a structural ultrasound. With this study and further steps for implementation of PRMs in maternity care, we are aiming to change the practice for even more PCCs by focusing on the areas of the needs of women and their families. This way, we can have more individual and value-based care. This research gathered experts’ or stakeholders’ opinions on a particular topic and can help implement PRMs into clinical practice. The results of this study underscore the significance of maintaining thorough and uniform focus on diverse PRMs within both public maternity care centers and hospital maternity environments, thereby guaranteeing a comprehensive and impactful approach to maternal healthcare.

### Strength, limitations, and future study

This study marks the first of its kind aiming to explore the primary responsible organization in public maternity healthcare for managing PRMs. Our approach involved a mixed-method survey administered to maternity healthcare professionals. The study’s strength lies in its diverse participant pool, encompassing professionals from all aspects of maternity healthcare—public maternity care centers, maternity clinics, delivery wards, and maternity wards, representing various occupations. A key advantage of this research is its contribution to a comprehensive understanding of healthcare professionals’ consensus on addressing PRMs in both public maternity care centers and hospital maternity settings. Emphasizing the consensus among participants, the study highlights public maternity care centers and hospital maternity wards as the primary organizations to respond in case assistance is needed. This insight benefits healthcare professionals, policymakers, and maternity care givers, providing specific areas of focus and consensus points. It aims to foster comprehensive and consistent attention to various PRMs, ensuring holistic and effective maternal care. To enhance transparency, the presentation of results includes textual and tabular representations, utilizing numerical data and percentages to offer a preliminary overview of professionals’ perspectives on the main providers of PRMs. While the study acknowledges a limitation in the lower participation from nurses working in the public maternity care centers (17) compared to midwives in the hospital (30), we believe this is compensated by the closer average number of nurses working in different units (public maternity care centers and the maternity clinic, delivery ward, and maternity ward). It is essential to note that this research was conducted exclusively within a specific health district in Finland. Therefore, the findings may not be universally applicable to all hospitals in Finland or relevant to professionals in different countries. Our future research aims to empirically investigate the implementation process, practices, and experiences related to electronic patient-reported measures (ePRM) in Helsinki University Hospital’s maternity care. We also intend to systematically assess the genuine benefits and value of incorporating ePRMs in operational development. The following research contributes to a more profound comprehension of the practical challenges surrounding ePRM adoption, advocating for evidence-based development and knowledge management in healthcare services. It will offer valuable insights into the systematic collection, utilization, and integration of quality metrics with other data. Our goal is to implement ePRMs nationwide in Finland’s maternity care.

## Conclusion

This study establishes a consensus that widely accessible public maternity care centers in Finland should be the primary organizations responsible for addressing PRMs in maternity care. The findings highlight crucial aspects such as *confidence with the role as a mother*, *maternal confidence with breastfeeding*, *satisfaction with the result of care*, *confidence in healthcare providers*, and *birth experience*, which should be addressed in both public maternity care centers and hospital maternity wards. Additionally, *background information* and *health-related quality of life* are considered important across organizations, with unanimous agreement for public maternity care centers. The study underscores the need for a standardized approach to ensure holistic and effective maternal care, emphasizing the importance of comprehensive attention to PRMs across public maternity care centers and hospital maternity settings.

## Electronic supplementary material

Below is the link to the electronic supplementary material.

Supplementary Material 1

## Data Availability

Data and material will be made available upon request by researchers.

## Abbreviations

PCC: Patient-centered care
VBHC: Value-based healthcare
ICHOM: International Consortium for Health Outcomes Measurement
PRMs: Patient-reported measures
PCB: Pregnancy and childbirth
PROMS: Patient-reported outcome measures
PREMS: Patient-reported experience measures
HRQoL: Health related quality of life
ePRM: Electronic patient-reported measures
